# Implications of stress-induced genetic variation for minimizing multidrug resistance in bacteria

**DOI:** 10.1186/1741-7015-10-89

**Published:** 2012-08-13

**Authors:** Uri Obolski, Lilach Hadany

**Affiliations:** 1 Department of Molecular Biology and Ecology of Plants, Tel Aviv University, Ramat Aviv, Tel Aviv 69978, Israel

**Keywords:** stress induced mutagenesis, HGT, antibiotic resistance, evolution, mathematical model

## Abstract

**Background:**

Antibiotic resistance in bacterial infections is a growing threat to public health. Recent evidence shows that when exposed to stressful conditions, some bacteria perform higher rates of horizontal gene transfer and mutation, and thus acquire antibiotic resistance more rapidly.

**Methods:**

We incorporate this new notion into a mathematical model for the emergence of antibiotic multi-resistance in a hospital setting.

**Results:**

We show that when stress has a considerable effect on genetic variation, the emergence of antibiotic resistance is dramatically affected. A strategy in which patients receive a combination of antibiotics (combining) is expected to facilitate the emergence of multi-resistant bacteria when genetic variation is stress-induced. The preference between a strategy in which one of two effective drugs is assigned randomly to each patient (mixing), and a strategy where only one drug is administered for a specific period of time (cycling) is determined by the resistance acquisition mechanisms. We discuss several features of the mechanisms by which stress affects variation and predict the conditions for success of different antibiotic treatment strategies.

**Conclusions:**

These findings should encourage research on the mechanisms of stress-induced genetic variation and establish the importance of incorporating data about these mechanisms when considering antibiotic treatment strategies.

## Background

Bacterial resistance to antibiotics has accompanied the introduction of new antibiotics since shortly after penicillin was first introduced [1] and is currently considered a major health issue [2,3]. The implications of infection with antibiotic-resistant bacteria include increased mortality rates, prolonged hospitalization and higher cost of treatment [1,4,5]. A particularly dangerous prospect of the continued evolution of drug resistance in bacteria is the creation of new, multidrug resistant bacteria. Such bacterial strains are already present in several species of bacteria [3,6] and treating them is more difficult and often accompanied by a period of ineffective treatment, resulting in increased patient mortality [6]. Moreover, the rate of new drug development is declining, leaving few treatment alternatives for treating the increasing burden of multi-resistant bacteria [7,8]. Since resistance is especially prevalent in hospitals [9], various treatment strategies have been suggested to facilitate better responses to resistant infections and minimize the emergence of new multi-resistant bacteria.

Three prominent strategies of antibiotic treatment are cycling, mixing and combining. Under a cycling regime, all the patients are treated with the same antibiotic drug at a given time, and the drug used is periodically switched. The rationale behind cycling is that each time an alteration of drugs is administered, the pathogens resistant to the previously used drug are attacked and are hopefully susceptible to the new drug [10]. In the mixing strategy, each patient receives a randomly selected drug. This strategy can be viewed as the default antibiotic usage within a hospital unit, when there is no preference for any particular antibiotic. In such a case, if two relevant antibiotics exist, approximately half the patients receive each drug at any given time. Mixing has the advantage of creating a heterogeneous stress environment for the bacterial population [10]. At each transmission, a bacterium has a probability of half encountering a drug to which it has not recently been exposed, and hence to which it is unlikely to be resistant. Combining is the administration of several drugs to each patient. By applying several antibiotics at once, combining is designed to diminish the chance of evolving resistance by eradicating any bacteria resistant to just one type of antibiotics. As a result, more antibiotics are used in combining than in mixing or cycling. This could lead to higher antibiotic-related toxicity and increased treatment costs [11].

Attempts to compare the different treatment strategies and assess their relative efficiency have been made both in empirical studies [12-17] and using theoretical analysis [10,18-20]. All in all, results obtained using both approaches have been inconclusive. It seems clear that current models do not capture all the aspects of the phenomenon, thus failing to properly distinguish between scenarios favoring different treatment strategies. We suggest that part of this shortcoming may result from the simplifying assumption that resistance is acquired at a constant rate, ignoring recent evidence to the effect of environmental stress on the mechanisms of resistance acquisition. The frequency of horizontal gene transfer (HGT) and mutation was shown to increase when bacteria are under various stressors, including nutritional deprivation, DNA damage, temperature shift, oxidative stress and exposure to antibiotics [21-24]. An environmental stressor especially relevant to our subject of inquiry is antibiotics. In *Streptococcus uberis*, acquisition of rifampin resistance through mutation was shown to increase more than 1,000-fold under ciprofloxacin [25]. Interestingly, the rifampin resistant mutants showed no resistance for ciprofloxacin, which indicates that this was not merely the result of selection. It was also shown that *Pseudomonas aeruginosa *increases its mutation rate by up to 10^5 ^in the presence of tetracycline antibiotics, and consequently obtains resistance to antibiotics [26]. Stress-induced mutation (SIM) might therefore have a substantial influence on the dynamics of antibiotic resistance acquisition [27-29]. In the context of HGT, *Streptococcus pneumoniae *was shown to increase the rate of chromosomal DNA uptake by transformation of a marker conferring resistance to streptomycin, when treated with either streptomycin or norfloxacin [30]. It was also shown that ciprofloxacin induces the transfer of the SXT integrating conjugative element, which is known to encode for antibiotic resistance genes, in SXT-containing *Escherichia coli *and in *Vibrio cholerae *up to 300-fold[31]. Phages were also observed to increase horizontal transfer of genetic material as a reaction to their host's antibiotic-induced SOS response[32], a process which might lead to an increased rate of antibiotic resistance acquisition [33]. Theoretical work also supports stress-induced genetic variation as a successful evolutionary strategy [34-36] so this phenomenon might be even more widespread then we currently know. Our goal is to explore the impact of genetic variation induced by antibiotic stress on the spread of antibiotic multi-resistance in a hospital unit. We use a classical modeling approach (first described in [37]), modified to describe SIM and stress-induced HGT. Our model is used to evaluate the efficacy of each treatment strategy under different assumptions regarding the effect of stress on genetic variation. We find that stress-induced variation can indeed alter the preferred treatment strategy.

## Methods

Our mathematical model describes the dynamics of bacterial infections in a hospital unit. The bacterial pathogens in question are assumed to accompany other ailments and not be the main reason for hospitalization. We consider two different antibiotic drugs, denoted antibiotic 1 and antibiotic 2. The frequencies of patients infected with bacteria resistant to antibiotics 1 and 2 are *R*_1 _and *R*_2 _, respectively, and the frequency of patients infected by susceptible bacteria is S. The frequency of uninfected patients is *X *. Clearance due to antibiotic usage occurs at rate *τ *, and χ*_i _*determines the fraction of patients receiving antibiotic i. Resistance is assumed to be complete, so that a patient infected by a bacterial strain resistant to drug 1 will not be affected at all by treatment with that drug. Conversely, if treated with drug 2, the patient becomes uninfected ( *X *) at rate *τ . γ *is the rate of spontaneous clearance due to the response of the patient's immune system, *β *is the rate of bacterial transmissions resulting in infection (for simplicity, superinfection is neglected), and *m *is the rate of patient turnover (so that the mean patient hospitalization time is $\frac{1}{m}$ days). Since we assume the bacterial infection is not the main reason for hospitalization, patients leave the hospital or die at a rate proportional to their frequency. The proportion of infected patients entering the hospital is determined by $\lambda_{R_{1}},\;\lambda_{R_{2}},\;\lambda_{S}$ for patients carrying bacteria resistant to antibiotic 1, to antibiotic 2, or to none, respectively. Uninfected patients enter the hospital at rate $m\left(1-\left(\lambda_{R_{1}}+\;\lambda_{R_{2}}+\;\lambda_{S}\right)\right)$, so hospital occupancy is kept constant. All of the parameters representing rates are given in units of day^-1^. We assume there are no double-resistant bacteria in the hospital initially, and that their frequency in the general population is negligible. This scenario may reflect situations where newly developed antimicrobial agents have been recently introduced, or were kept as the last resort, so that double resistance is still scant.

These parameters are incorporated in the following set of ordinary differential equations:

(E1) $$\begin{array}{c} \frac{dX}{dt}=\left(1-\lambda_{S}-\lambda_{R_{1}}-\lambda_{R_{2}}-X\right)m+\left(\tau +\gamma \right)S+\left(\tau \chi_{2}+\gamma \right)R_{1}\\ +\left(\tau \chi_{1}+\gamma \right)R_{2}-\beta X\left(S+R_{1}+R_{2}\right)\\ \frac{dS}{dt}=\left(\lambda_{S}-S\right)m-\left(\tau +\gamma \right)S+\beta XS\\ \frac{dR_{1}}{dt}=\left(\lambda_{R_{1}}-R_{1}\right)m-\left(\tau \chi_{2}+\gamma \right)R_{1}+\beta XR_{1}\\ \frac{dR_{2}}{dt}=\left(\lambda_{R_{2}}-R_{2}\right)m-\left(\tau \chi_{1}+\gamma \right)R_{2}+\beta XR_{2} \end{array}$$

**Figure 1:**
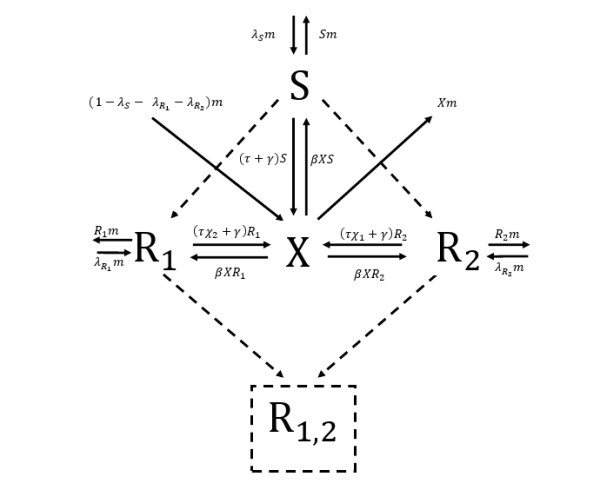
**Illustration of antibiotic resistance dynamics in a hospital setting**. The solid lines represent infection, recovery and patient turnover. Dashed lines represent the effects of HGT and mutation. Stress-induced genetic variation would result in an increased weight of the dashed lines under antibiotic stress. *X*, *S*, *R*_1_, *R*_2 _are the frequencies of uninfected patients, patients infected with susceptible bacteria, and patients with bacteria resistant to antibiotics 1 and 2, respectively. They enter the hospital with rates $\left(1-\lambda_{s}-\lambda_{R_{1}}-\lambda_{R_{2}}\right)m,\lambda_{s}m,\lambda_{R_{1}}m,\lambda_{R_{2}}m$, and leave with a rate proportional to their frequency, where the patient turnover rate, *m *, is the proportion constant. Infected patients turn uninfected either through spontaneous recovery due to the immune system (at rate *γ*), or due to antibiotic treatment (at rate *τ*). *χ*_1 _and *χ*_2 _determine which amount of antibiotics 1 and 2 are used, respectively. Uninfected patients become infected at rate *β *multiplied by the frequencies of cleared and infected patients. *R*_1,2 _denotes the fraction of patients infected with double resistant bacteria, assumed to be zero at the beginning. HGT, horizontal gene transfer.

The equations describe the rate of change of patient frequencies within a hospital. The dynamics are illustrated in Figure 1.

Equations E1 were solved analytically [See Additional file 1, section 3], and by numerical integration using Matlab^® ^R2009a.

Moving average calculation: We used the numerical solutions of equations E1 and the analytical computations of double resistance emergence [See Additional file 1, sections 1 and 2] to calculate values of a moving average. First, the different sets of parameters are ordered according to the parameter plotted on the × axis. Each point in the plot presents the average double resistance emergence over 201 equally weighted parameter sets: the one corresponding to the point itself, the 100 nearest parameter sets with lower values of $\tau \left(\frac{\lambda_{R_{1}}}{\lambda_{R_{2}}}\right)$, and the 100 nearest parameter sets with higher values of$\tau \left(\frac{\lambda_{R_{1}}}{\lambda_{R_{2}}}\right)$. The same 201 data sets are used to calculate double resistance emergence for a given value of $\tau \left(\frac{\lambda_{R_{1}}}{\lambda_{R_{2}}}\right)$ under each of the three strategies, resulting in a correlation between the three values, and a similarity in the shape of the three moving average curves (since the three means are taken over the same parameter sets).

## Results

### Stress-induced mutation

Considering stress-induced mutation, we define *μ_s _*and *μ_r _*as the rates of mutations conferring antibiotic resistance when bacteria are under antibiotic stress and when they are free of antibiotic stress, respectively. We assume that mutation is increased by stress, that is, *μ_s _*>*μ_r_*. Our work concentrates on the scenario where sensitive bacteria (corresponding to host frequency *S *) have a negligible contribution to the generation of single resistant bacteria. In terms of our dynamics, this translates to the following inequality:

(C1) $$\mu_{s}S\ll \lambda_{R_{l}}m+\beta XR_{i},i=1,2$$

Intuitively, inequality C1 is satisfied due to either abundance of single resistance in the population outside the hospital, causing high entrance rates of single resistant bacteria, or abundance inside the hospital due to infection and selection.

An important feature of antibiotic resistance is its persistence within a host without direct selective forces for long periods of time [38]. Direct selection occurs when a patient is treated with a certain antibiotic, say antibiotic 1, and a new bacterium resistant to antibiotic 1 arises within the host, by mutation or HGT. Thus, due to strong selection for antibiotic resistance, it has a high probability of taking over the entire infecting population and turning the host to *R*_1_. When the host is not treated with antibiotic 1, resistance to antibiotic 1 might not confer any direct fitness advantage. Thus, we assume that the probability of a bacterium to take over the infection in the second scenario is *σ *times the chance in the first scenario, where 0 <*σ *< 1 represents the relative persistence of antibiotic resistance when there is no direct antibiotic usage. Under condition C1, we can consider *σ *only when computing within-host dynamics of double resistant bacteria.

The exact value of *σ *is hard to measure, but is likely to be non-zero, as evidence suggests that patients who have not been treated with antibiotics for periods of up to three years still carry antibiotic resistant bacteria [6] . One reason for high persistence of resistant bacteria in ambulatory patients and medical staff might be residuals of antibiotics that are found in the environment at amounts sufficient to change the fitness of sensitive bacteria. This might often be the case in hospitals, as it was shown that even very low concentrations of antibiotics can select for antibiotic resistant bacteria [39] and that even ambulatory patients who have not received antibiotics for long periods of time harbor high frequencies of antibiotic resistant bacteria [40].

To compute the emergence of double resistance through mutation we first define a term describing the sum of the frequencies of patients carrying single resistant bacteria in the hospital for a certain time period:

$$E_{SIM}\left(U\right):=\overset{t_{1}}{\underset{t_{0}}{\int }}\left(R_{1}\left(\text{U}\right)+R_{2}\left(\text{U}\right)\right)dt$$

Using *E_SIM _*and the parameters described above, we can now write a term describing the emergence of double-resistant bacteria under treatment strategy *U*. It will be denoted by *ξ_SIM _*(*U *). Writing *ξ_SIM _*explicitly for the three strategies results in the following expressions [See Additional file 1, section 2, equations 7, 8, 10]:

$$\xi_{SlM}\left(mixing\right)=\frac{1}{2}\left(\mu_{s}+\mu_{r}\sigma \right)E_{SlM}\left(mixing\right),$$

$$\xi_{SIM}\left(combining\right)=\mu_{s}E_{SIM}\left(combining\right)$$

**Figure 2:**
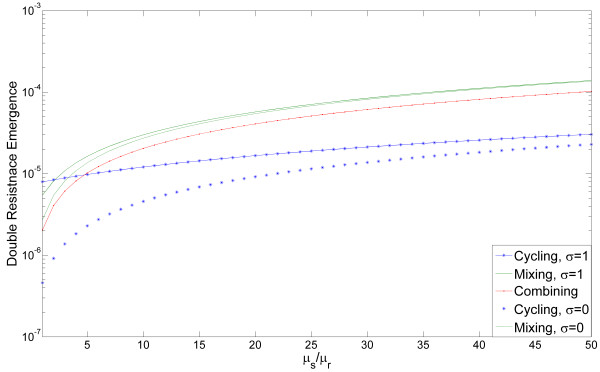
**Emergence of double resistance as a function of the effect of stress on mutation**. The double resistance emergence for each strategy is plotted, in log scale, as a function of $\frac{\mu_{s}}{\mu_{r}}$, which varies from 1 to 50. Note that lower emergence values indicate higher efficiency of the strategies. Mixing is plotted in green, cycling in blue, and combining in red. Double resistance emergence is monotonically increasing in *σ *(persistence of antibiotic resistance), so curves with intermediate values of *σ *will be within the boundaries of the curves *σ *= 0 and *σ *= 1 displayed. The value of *σ *is irrelevant for combining, so only one curve is produced. An intersection of the curves implies that the preference between two strategies, with respect to the emergence of double resistance, should change. We can see that for a wide range of $\frac{\mu_{s}}{\mu_{r}}$, cycling is the most efficient strategy. When *σ *is low enough, cycling is the most efficient strategy even without SIM. The emergence is taken over 600 days. The length of each cycle in the cycling strategy is 200 days. Other parameter values: *β *= 0.9, *γ *= 0.03, *m *= 0.1, *λ_s _*= 0.1, $\lambda_{R_{1}}=0.1$, $\lambda_{R_{2}}=0.1$, *τ *= 0.5. SIM, stressed-induced mutation.

For cycling we have a more complex expression. We will divide time into segments in which only one antibiotic is applied. In each of these segments only one strain of resistant bacteria is under antibiotic stress. Thus,

*ξ_SIM _*(*cycling *) = *μ_s_A + μ_r _σ B *where *A *= ∫ *R_i _*(*cycling*) when antibiotic *j ≠ i *is applied and *B = ∫ R_i _*(*cycling*) when antibiotic *i *is applied [See Additional file 1, section 2, definition (9)]. This allows us to prove that as $\frac{\mu_{s}}{\mu_{r}}$ increases, emergence of double resistance in cycling decreases relative to the emergence of double resistance in mixing and combining [See Additional file 1, section 2.1, equations 11-12]. This is shown in Figure 2, where emergence of double resistance by mutation for each strategy is plotted as a function of $\frac{\mu_{s}}{\mu_{r}}$. Dashed and solid lines correspond to *σ = *0 and *σ = *1. Double resistance emergence is monotonically increasing in *σ *[See Additional file 1 section 2, equations 7, 10], thus the values for intermediate values of *σ *are between the two curves. Some intuition about the relative success of cycling can be obtained from looking at the fraction of stressed bacteria under each strategy: under the combining strategy, all bacteria are under antibiotic stress; under the mixing strategy half of the bacteria of each resistant strain will be under antibiotic stress; cycling has the advantage that a resistant strain of bacteria will be under antibiotic stress only at certain periods of time - when all patients are receiving the antibiotic to which this strain is sensitive. During these periods the hyper-mutating strain would typically be at low frequency. This causes the increase in double resistance emergence due to SIM to be more moderate under cycling than under mixing or combining. Persistence of antibiotic resistance,*σ *, also has a strong influence on the dynamics: *σ *and *μ_r _*appear in our equations only within a single term *σ · μ_r _*, thus low values of *σ *and high values of $\frac{\mu_{s}}{\mu_{r}}$ have similar effects on the dynamics. The special case where we assume no stress induction and *σ *= 0 was previously studied by Bonhoeffer *et al*. [19] and our results are consistent with theirs. However, our conclusions hold for intermediate values of *σ *as well, assuming mutation rates are sufficiently dependent on stress.

In addition to the parameters pertaining to the stress induction mechanisms and within-host selection, the parameters determining *E_SIM _*also play an important role in the emergence of double resistance. To test the robustness of our model to changes in these parameters we study the emergence of double resistance for 10^4 ^random sets of parameters. The values of *m *were chosen from a generalized extreme value distribution fitted to the length of patients' stay in observed data [41]. Antibiotic clearance rates, $\frac{1}{\tau }$, were chosen from a uniform distribution on [2,14] (deduced from optimal treatment estimations in [42]). Spontaneous clearance, *γ*, is then chosen from a uniform distribution on [0,*τ*] , and Infection rates, *β*, were chosen from a log uniform distribution on [0.001,1] [10]. Four more values were chosen from the uniform distribution on 0[1], and then normalized to determine the entrance frequencies $\lambda_{s},\lambda_{R_{1}},\lambda_{R_{2}},1-\lambda_{s}-\lambda_{R_{1}}-\lambda_{R_{2}}$.

**Figure 3:**
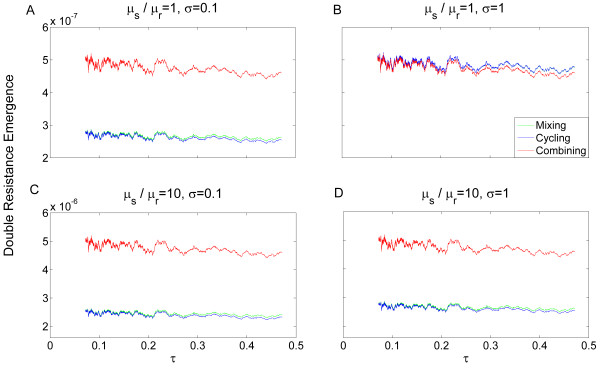
**Robustness of the dynamics describing resistance acquisition by mutation**. The dynamics describing double resistance emergence by mutation were studied for 10^4 ^random sets of parameters (derived from the distributions described in the main text). The moving average of the emergence of double resistance (see methods) is plotted as a function of the rate of clearance due to antibiotic usage (*τ*). Note that the same data sets are used for a given value of *τ *under each of the three strategies, generating an association between the curves. The moving average of mixing is plotted in green, of cycling in blue, and of combining in red. When the values of the moving average for mixing and cycling are very close only the blue curve is visible. (**A**) $\frac{\mu_{s}}{\mu_{r}}$ = 1, *σ *= 0.1. When *σ *is low, cycling decreases the emergence of double resistance better than the other strategies even with no influence of stress on mutation rates. (**B**) $\frac{\mu_{s}}{\mu_{r}}$ = 1, *σ *= 1. When *σ *is high and stress has no effect on mutation, combining is more efficient than the other strategies. (**C**) $\frac{\mu_{s}}{\mu_{r}}$ = 10, *σ *= 0.1. At low *σ*, increased influence of stress on mutation rates results in increased emergence by about an order of magnitude (compare with A), where cycling remains the most efficient strategy. (**D**) $\frac{\mu_{s}}{\mu_{r}}$ = 10, *σ *= 1. At high *σ*, increase in mutation rates due to stress alters the preferred strategy drastically from combining to cycling (compare with B).

In Figure 3 we plot the value of a moving average (see Methods) of the emergence of double resistance, where the horizontal axis is taken as the rate of clearance due to antibiotic usage (*τ *). The moving average of double resistance under mixing is plotted in green, under cycling in blue, and under combining in red. When the values of the moving average for mixing and cycling are very close, only the blue curve is visible. Two values of the persistence of antibiotic resistance,*σ*, are presented: *σ *= 0.1(panels A, C) and *σ *= 1 (panels B, D). Cycling outperforms the other strategies given even a moderate influence of stress on mutation rates, or a low enough value of *σ*. As *τ *increases, combining becomes slightly more effective (as it reduces single resistance frequency efficiently) but the effect is weak. Whenever mutation is stress-induced, or *σ *has a low value, combining would be the least efficient strategy in terms of inhibiting emergence of double resistance.

**Figure 4:**
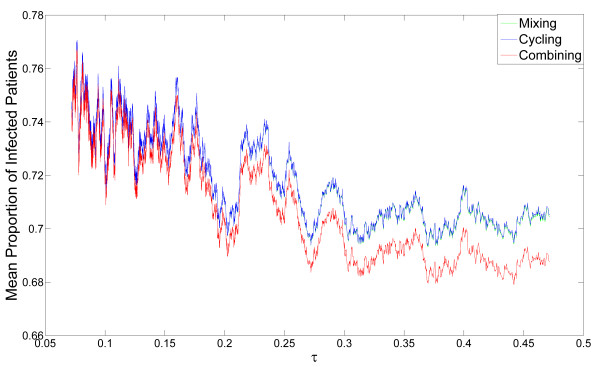
**Robustness of the dynamics describing proportion of infected patients**. The dynamics described in E1 were studied for 10^4 ^random sets of parameters (derived from distributions described in the main text). The moving average of the emergence of double resistance (see Methods) is plotted as a function of the rate of clearance due to antibiotic usage (*τ*). Note that the same data sets are used for a given value of *τ *under each of the three strategies, generating an association between the curves. The moving average of mixing is plotted in green, of cycling in blue, and of combining in red. When the values of the moving average for mixing and cycling are very close, only the blue curve is visible. We can see that combining is the most efficient strategy in reducing the proportion of infected patients. When the rate of clearance due to antibiotics, *τ*, increases, the relative efficiency of combining increases , while the proportion of infected patients decreases.

Different results are obtained when observing the mean proportion of infected patients rather than double resistance emergence. Figure 4 shows the mean frequency of infected patients in the hospital, for the same random sets of parameters as in Figure 3 (note that under condition C1, any changes in parameters affecting stress-induction are irrelevant). We can see that combining always outperforms cycling and mixing in terms of minimizing overall infected patients. When antibiotic resistance persistence,*σ *, is high and mutation is not stress-induced, combining reduces both infection and emergence more efficiently than the other strategies (Figure 3.B), but only by a few percent. However, under stress-induced mutation, or low values of *σ*, there is a conflict between minimizing infection and minimizing emergence of resistance: combining reduces the proportion of infected patients by about 1% on average, but increases emergence of double resistance by more than 50% (see Figure 3 and Additional file 1 table S1). Similarly, when *σ *is high and mutation is not stress-induced, mixing slightly reduces both infection and emergence in comparison with cycling (Figure 3.B and Additional file 1, table S1). Under stress-induced mutation, or low values of *σ*, there is a conflict: mixing reduces the proportion of infected patients (by about 0.1% on average), but increases emergence. For a large subspace of realistic parameter sets [43] double resistance is substantially decreased under cycling, relative to mixing, under the assumption of SIM [See Additional file 1, Figure S1].

The clearance rate due to antibiotic usage,*τ*, mildly decreases both infection (Figure 4) and resistance emergence (Figure 3). As the clearance rate increases, bacteria are eliminated more efficiently, thus the window of opportunity for infecting bacteria to acquire double resistance narrows.

### Stress-induced horizontal gene transfer

Another mechanism for acquiring antibiotic resistance is HGT. Acquiring antibiotic resistance through HGT is a process that depends on the rate of encounters between bacteria of different resistant strains, and on the probability of bacteria to donate and receive genetic material. Bacteria of one strain will encounter bacteria of another strain at a rate proportional to the amount of interactions between patients infected by these bacteria. Hence, we define

$$E_{HGT}\left(U\right):=\overset{t_{1}}{\underset{t_{0}}{\int }}R_{1}\left(U\right)R_{2}\left(\text{U}\right)dt$$

For the rate of encounters between bacteria of different resistant strains we multiply *E_HGT_*(*U) *by a constant C, which denotes the rate of bacterial transmission from one patient to another (by hospital staff, direct contact, and so on). We denote by *d *the probability that bacteria will donate genetic material, and by *r *the probability that bacteria will receive genetic material, given that a bacterial transmission event has occurred. The probability of successful HGT between bacteria, given bacterial transmission, is thus *r *· *d *. However, when stress-induced HGT is considered, *r *and *d *are no longer constant, as different treatment strategies create varying levels of stress for different bacteria. We will define *r_r_*, *d_r _*to be the probabilities of receiving and donating genetic material for bacteria which are not under antibiotic stress. Similarly, *r_s _*and *d_s _*will be the probabilities of receiving and donating genetic material for bacteria which are under antibiotic stress. For example, if all patients are treated with the same drug, bacteria resistant to that drug would perform HGT with probabilities *r _r _*and *d _r _*, while bacteria sensitive to it would perform HGT with probabilities *r _s _*and *d _s _*. Stress-induced HGT will be expressed by the conditions *r _s _*>*r _r _*and *d _s _*>*d _r _*.

Note that when different patients are treated with different types of antibiotics, bacteria may be transported from a non-stressful environment to a stressful one and vice versa. *ϕ *will represent the time required for the cellular mechanisms to induce or repress HGT. Namely, when *ϕ *= 0 a change in the bacteria's well-being is immediately translated to a change in HGT rates, whereas for larger values of ϕ the stress-induction mechanisms react more slowly to changes in environmental stress. We define the extreme case of *ϕ *= 1 as the case where the HGT rates of bacteria transmitted from patient A to patient B depend only on the stress the bacteria experienced while residing in patient A. In analogy to inequality (C1), we concentrate on the case where the influence of non-resistant bacteria on the emergence of double resistance is negligible. This can be formally expressed as:

(C2) $$Cd_{s}r_{s}SR\ll \lambda_{R_{l}}m+\beta XR_{i},i=1,2$$

Using the parameters and assumptions described above, we denote by *ξ_HGT _*(*U*) the emergence of double-resistant bacteria through stress-induced HGT under treatment strategy *U*, and derive *ξ_HGT _*for each of the strategies [See Additional file 1, section 1, equations 1-3]. The combining strategy is the simplest to model, since all patients are treated with at least one effective antibiotic. Therefore, the emergence of double resistance under combining is

$$\xi_{HGT}\left(combining\right)=Cr_{s}d_{s}E_{HGT}\left(combining\right).$$

Emergence under cycling can be broken into time intervals in which only one drug is used. Assuming there is an equal amount of such intervals for each drug we get

$$\xi_{HGT}\left(cycling\right)=C\frac{1}{4}\left(r_{r}d_{s}\sigma +r_{s}d_{r}\sigma +r_{r}d_{s}+r_{s}d_{r}\right)E_{HGT}\left(cycling\right).$$

Note that for both combining and for cycling, *ϕ *does not appear in the term describing double resistance emergence, since under both strategies bacteria will not experience an environmental change when transported from one patient to another. For mixing, the heterogeneity of the environment due to the usage of different antibiotics requires the use of *ϕ *:

$$\begin{array}{c} \xi_{HGT}\left(mixing\right)=C\frac{1}{8}[r_{s}\left(d_{s}\phi +d_{r}\left(1-\phi \right)\right)+r_{r}d_{s}\sigma +r_{s}d_{r}+r_{r}\left(d_{r}\phi +d_{s}\left(1-\phi \right)\right)\sigma +\\ d_{s}\left(r_{s}\phi +\left(1-\phi \right)r_{r}\right)+d_{s}r_{r}+d_{r}r_{s}\sigma +d_{r}\left(r_{r}\phi +r_{s}\left(1-\phi \right)\right)\sigma ]E_{HGT}\left(mixing\right) \end{array}$$

Analysis of *ξ_HGT _*for each strategy shows that high enough values of min $\left\{\frac{d_{s}}{d_{r}},\frac{r_{s}}{r_{r}}\right\}$will cause the ratio $\frac{\xi_{HGT}\left(cycling\right)}{\xi_{HGT}\left(combining\right)}$ to be arbitrarily close to zero [See Additional file 1, section 1.1, equation (6)]. In other words, the more HGT becomes affected by stress in both the donor and the recipient ends, the more efficient cycling becomes relative to combining. This occurs due to the fact that when cycling is applied only one type of resistant bacteria is stressed at any given time. For double resistant bacteria to emerge through HGT, a donor and a recipient of different resistance types are needed, but under cycling they will never be simultaneously under stress. In contrast, when the combining strategy is applied all bacteria are stressed, including potential donors and recipients alike.

The efficiency of the mixing strategy depends on *ϕ*. This results from the fact that under mixing, different patients are treated with different drugs. When *ϕ *is low, bacteria transported from one patient to another change their HGT probabilities according to the drug taken by the new host. Therefore, for both mixing and cycling, bacterial HGT events which produce double resistance will rarely occur when both donor and recipient are stressed. In contrast, when *ϕ *is high, bacteria transported from one patient to another retain HGT probabilities complying to the antibiotic treatment of their former host, allowing the emergence of double resistance through HGT in which both donor and recipient are stressed.

When exploring the effects of stress induced HGT we take $\frac{d_{s}}{d_{r}}=\frac{r_{s}}{r_{r}}$, since we have shown that min $\left\{\frac{d_{s}}{d_{r}},\frac{r_{s}}{r_{r}}\right\}$ is the dominant factor in the dynamics [See Additional file 1, section 1.1 equations 4-6], and in order to avoid assumptions about the role of donor and recipient in the dynamics. For ease of notation we define $\theta :=\frac{d_{s}}{d_{r}}=\frac{r_{s}}{r_{r}}=\text{min}\left\{\frac{d_{s}}{d_{r}},\frac{r_{s}}{r_{r}}\right\}$.

**Figure 5:**
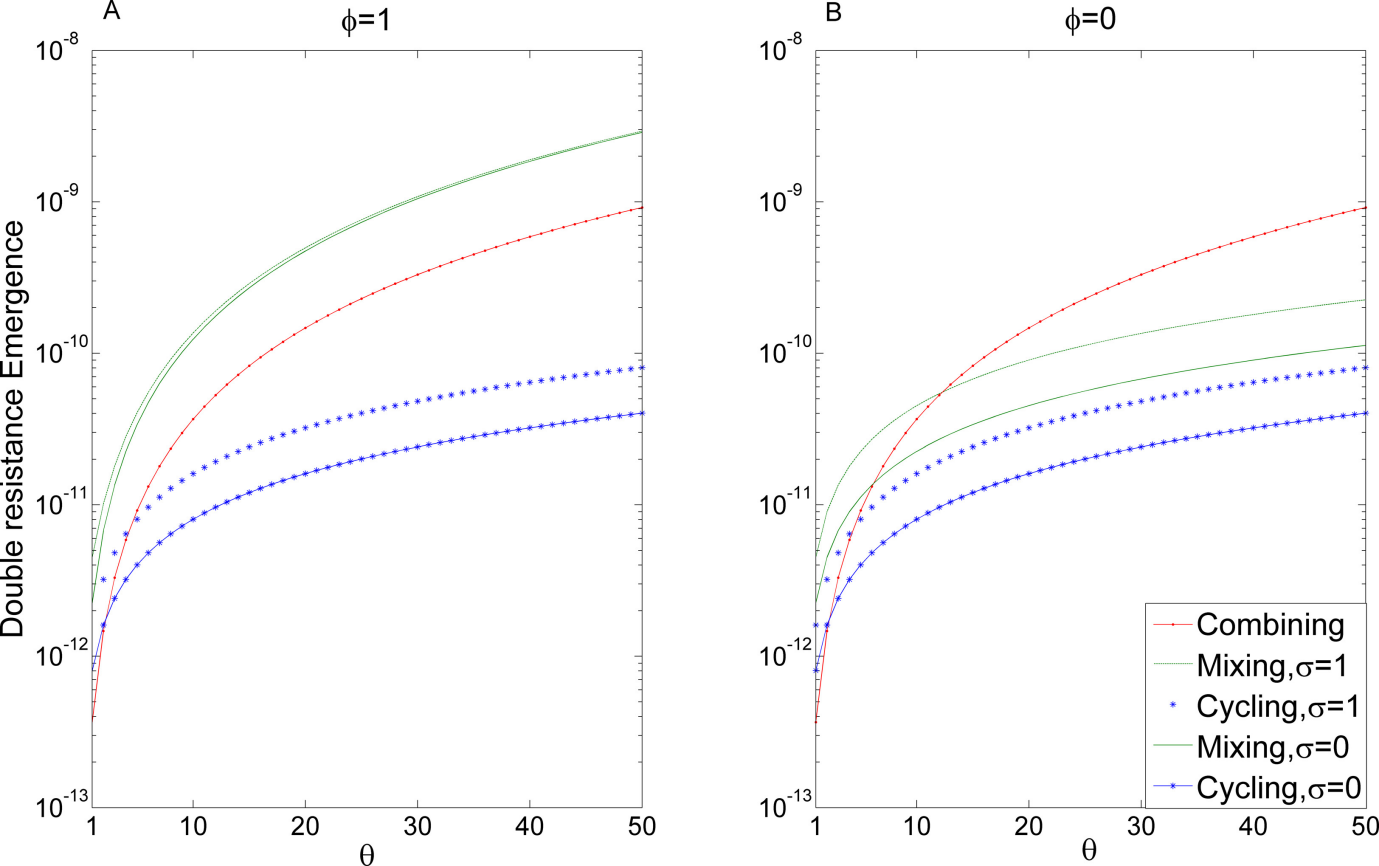
**Emergence of double resistance as a function of the effect of stress on HGT**. Double resistance emergence for each strategy is plotted, in log scale, as a function of *θ*, the effect of stress on HGT. Mixing is plotted in green, cycling in blue, and combining in red. We see that stress-induced HGT changes the efficiency of the strategies so that cycling is the most efficient strategy for a wide range of *θ *values - note that lower emergence indicates a more efficient strategy. Panel **A **shows results for *ϕ *= 1, whereas in panel **B ***ϕ *= 0. We can see that the double resistance emergence for mixing indeed increases dramatically for *ϕ *= 1 in comparison with *ϕ *= 0 , matching the intuitive explanation (see text). An intersection of the curves implies that the preference between two strategies should change. The mean is taken over 600 days, where the length of each cycle in the cycling strategy is 200 days. Other parameter values: *β *= 0.9, *γ *= 0.03, *m *= 0.1, *λ_s _*= 0.1, $\lambda_{R_{1}}=0.1$, $\lambda_{R_{2}}=0.1$, *τ *= 0.5, *C *= 1 (note that changing C will just rescale the horizontal axis, so its value is arbitrary). HGT, horizontal gene transfer.

Figure 5 shows the rate of double resistance emergence for each strategy as a function of *θ*. We can see that the rate of double resistance emergence depends also on *ϕ *(compare panels A and B of Figure 5), and to a lesser extent on *σ *(dashed and solid curves in each panel correspond to *σ *= 1 and *σ *= 0 , respectively).

**Figure 6:**
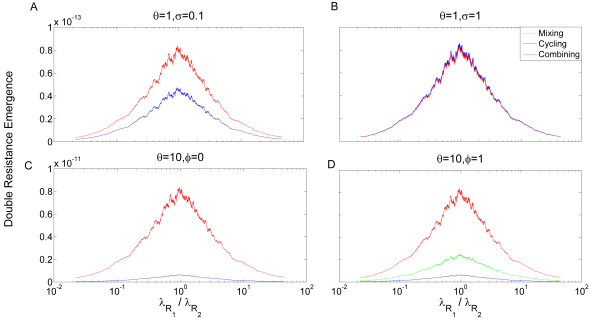
**Robustness of the dynamics describing resistance acquisition by HGT**. The dynamics describing double resistance emergence by HGT were studied for 10^4 ^random sets of parameters (derived from distributions described in the main text). The moving average of the emergence of double resistance (see Methods) is plotted as a function of the entrance rates' ratio ($\frac{\lambda_{R_{1}}}{\lambda_{R_{2}}}$). Note that the same data sets are used for a given value of $\frac{\lambda_{R_{1}}}{\lambda_{R_{2}}}$ under each of the three strategies, generating an association between the curves. The emergence rate under mixing is plotted in green, under cycling in blue, and under combining in red. When the emergence rates under mixing and cycling are very close, only the blue curve is visible. In (**A**) and (**B**), HGT is not affected by stress at all (*θ *= 1) so the delay of stress-induction mechanisms (*ϕ*) is irrelevant. In contrast, persistence of antibiotic resistance (*σ*) is influential in such cases, and we present two extreme values. (A) *θ *= 1,*σ *= 0.1. Cycling and mixing are the dominant strategies, where their relative efficiency decreases at asymmetric entrance rates. (B) *θ *= 1, *σ *= 1 . Combining is the preferred strategy for inhibiting double resistance emergence. In (**C**) and (**D**) HGT is stress induced with *θ *= 10, so while *ϕ *is now an influential parameter, the effect of *σ *is very minor (Additional file 1, section 1) and we present only the value *σ *= 0.5. (C) *θ *= 10, *ϕ *= 1, *σ *= 0.5 . Mixing is slightly more preferable than cycling, and cycling is most inefficient when entrance rates are asymmetric. (D) *θ *= 10, *ϕ *= 0, *σ *= 0.5 . Cycling is the preferred strategy for inhibiting double resistance emergence. HGT, horizontal gene transfer.

The ability of a strategy to minimize *E_HGT _*plays an important role in the emergence of double resistance through HGT as well. Since the dynamics determining *E_HGT _*are complex we will focus only on several important factors. First, we note that when the cycle length approaches zero, double-resistance emergence under cycling converges to double-resistance emergence under mixing, as was shown in [10]. Second, when there is strong asymmetry between the frequencies of different single resistant bacterial infections, cycling performs poorly. If the current drug used is ineffective against the bacterial strain with the high entrance rates it will allow the incidence of that strain to increase rapidly. This was shown by Bergstrom *et al*. [10], who predicted that under high asymmetry of single resistance entrance rates cycling would not be an efficient strategy. While this is true for a model assuming constant HGT, we argue that when HGT is stress-induced, and the response to environmental changes is not immediate (that is, *ϕ *> 0 ), cycling can still be the most efficient strategy. When the response is immediate, mixing will tend to minimize double resistance emergence best. Combining will rarely minimize double resistance better than the other two strategies when HGT is stress induced. These conclusions were obtained by studying two prominent factors that influence the emergence of double resistance: the ratio of entrance rates [See Additional file 1, section 3] and the effect of stress on HGT. We have randomly selected 10^4 ^parameter sets (the same parameter sampling as in Figure 3) and for each parameter set computed the moving average of the emergence of double resistance by HGT for the three strategies, as a function of entrance rates' ratio $\frac{\lambda_{R_{1}}}{\lambda_{R_{2}}}$ (in log scale). The moving average of mixing is plotted in green, of cycling in blue, and of combining in red. When the values of the moving average for mixing and cycling are very close, only a blue curve is visible. We can see that when HGT is completely unaltered by stress, the relative efficiency of the strategies depends on the persistence of antibiotic resistance:*σ *. When *σ *is high, combining is the best strategy at minimizing double resistance for most parameter sets (Figure 6.B). However, when *σ *decreases, mixing becomes a prominent strategy as well, with cycling close behind it (Figure 6.A). In Figure 6.C and 6.D, we allow mild stress induced HGT and take *θ *= 10. *σ *has a lesser influence when HGT is stress-induced [See Additional file 1, section 1.1] and, therefore, we set it at an intermediate value of *σ *= 0.5 . Cycling and mixing now become more efficient strategies than combining. We can also see the influence of *ϕ *on whether cycling or mixing will be the most efficient strategy in terms of inhibiting double resistance emergence (The influences of other parameters are shown in Figure S2 in Additional file 1).

As we mentioned above, combining always outperforms cycling and mixing in terms of minimizing overall infected patients, but the benefit is of only a few percent (Figure 4 and Additional file 1, table S1). When antibiotic resistance persistence,*σ*, is low, even without stress induction, emergence of double resistance under combining is higher by 30% than under other strategies [See Additional file 1, table S1]. When stress induction is significant, double resistance emergence under combining is more than tenfold higher than under cycling, and more than 30% higher than under mixing (Figure 6 and Additional file 1, table S1).

## Discussion

Several conclusions can be derived from our mathematical model. We have shown that stress-induced genetic variation can have a drastic influence on the emergence of double resistance, and should be considered when deciding on a hospital wide strategy of antibiotic usage. Although always slightly more efficient than other strategies in decreasing the incidence of single resistant infections, the strategy of combining performs very poorly in inhibiting double resistance emergence when genetic variation is stress-induced. This holds true despite the fact that under the combining strategy all patients receive effective treatment, and even though we disregard the toxic effects of combining antibiotics for the patient and the economic burden it carries for the population [11,44].

Cycling is the preferred strategy with respect to the acquisition of resistance through SIM. Low persistence of antibiotic resistance (*σ*) further amplifies the effects of SIM and increases the relative efficiency of cycling. In the presence of stress-induced HGT, cycling and mixing are the favored strategies, and the preference between them is determined by how fast the bacteria respond to environmental changes (the parameter *ϕ *in our model). If changes in HGT frequencies in response to antibiotic stress are rapid, mixing is the preferred strategy, whereas slow response to stress would tilt the scales in favor of cycling. We should note that our predictions hold even for a very mild increase of HGT and mutation rates under antibiotic stress (Figures 3 and 6) in comparison with those described in the literature [25,26,30,31]. Higher dependence of variation on stress leads to results which are more robust to changes in other parameter values.

There are several criteria which are used to evaluate the efficiency of an antibiotic strategy: reduction of total infection burden; single resistance minimization; and inhibition of multiple resistance emergence [10,19,20,43,45,46]. We compared two measures of treatment efficiency: proportion of infected patients and emergence of double resistance. The latter is of interest mainly in a population where double resistance bacteria are still at a low frequency, thus we focused on that scenario. There is rarely a strategy which is ideal for both infection and emergence of double resistance at the same time. A strategy that is successful at reducing infection applies more accurate treatment, and this has two effects on emergence: on the one hand, eliminating infection and thus minimizing the bacteria that would become resistant. On the other hand, treatment creates selective pressure and potentially stress induced variation - two factors that might lead to a faster generation of resistance. We find that combining always outperforms mixing and cycling by a small amount, when it comes to minimizing infection. When considering double resistance emergence, both mixing and cycling outperform combining substantially when variation is stress-induced. This contrast should be taken into account when deciding on a treatment strategy.

We make several assumptions that should be discussed explicitly. First, we did not consider the possible fitness cost of antibiotic resistance. This is consistent with recent evidence suggesting that compensatory mechanisms reduce such cost to a low level [27,47,48]. Additionally, for stress-induced HGT to have substantial influence on the dynamics we require that both donor and recipient probabilities of HGT would increase with stress. It was shown in [30] that acquisition of resistance through transformation increases with the recipient's stress. The amount of genetic material available for transformation in the environment is influenced by the death of bacteria, and is therefore dependent on the stress that the donor bacteria experience. This is particularly true when phages cause lysis of their host when the host is stressed [32,33]. Similarly, donation and acquisition of conjugative elements were each, separately, shown to increase under stress [31,49,50]. Another issue we did not address is the influence of stochastic events on the dynamics. The population size within a hospital unit might be small enough for stochastic events such as epidemic outbursts of bacteria and extinction of rare bacterial strains for long periods of time, to be very influential [45]. Human errors in the form of dosage errors, lack of compliance to hospital guidelines and so on can be another source of stochastic noise that might shift the dynamics from the deterministic expectation described here.

Furthermore, the values of certain parameters might be different for different patients. For instance, elderly patients might be more susceptible to bacterial infections than other patients [51,52]. This could be expressed by modeling compartments of patients with different parameter values (in this example, higher *β *values) in accordance with the epidemiological data. Finally, the relative efficiency of the different drugs was assumed to be equal, and no drug interactions were considered - two factors that may further affect the evolution of resistance [53]. Future work could address these matters explicitly.

Our model points to several directions in which empirical data can guide the planning of efficient treatment strategies. First, it is important to understand whether a pathogen acquires resistance primarily through mutation or through HGT. Second, it is important to estimate the persistence of antibiotic resistant bacteria within hosts not currently treated with antibiotics effective against those bacteria (the parameter *σ *in our model). Finally, we would like to directly assess the degree to which stress, and in particular antibiotic stress, increases the rates of bacterial mutation and HGT. Obtaining such data would be an important step in the ongoing struggle against multi-drug resistance. We believe that obtaining such precise data will help to decrease the prevalence of multiple resistance strains in bacterial pathogens which have already shown to increase genetic variation under stress, such as *P. aeruginosa, S. pneumoniae, E. coli *and *V. cholerae *[26,30,31].

## Conclusions

In conclusion, our work presents an important factor thus far overlooked when planning antibiotic treatment strategies, namely the effect of stress on genetic variation. We show that considering the effects of stress-induced genetic variation alters the results of existing theoretical models: specifically, combining antibiotics may result in an increased rate of emergence of double resistant bacteria, whereas cycling antibiotics can be more effective than previously thought. Applying our predictions to specific pathogens would require better empirical evaluation of a few key parameters that affect the dynamics of double resistance emergence. We make specific predictions regarding the parameter values that would favor particular treatment strategies, suggesting that further investigation of stress-induced variation and its mechanisms might have crucial importance for combating multiple antibiotic resistance.

## Abbreviations

HGT: horizontal gene transfer; SIM: stress-induced mutation.

## Competing interests

The authors declare that they have no competing interests.

## Authors' contributions

UO and LH conceived of the study and designed the model. UO performed the mathematical analysis, simulations, and data analysis. UO and LH wrote the paper. The authors read and approved the final manuscript.

## Pre-publication history

The pre-publication history for this paper can be accessed here:

http://www.biomedcentral.com/1741-7015/10/89/prepub

## Supplementary Material

Additional file 1 **Proofs and additional figures and tables**. Additional file 1 contains proofs for all the equations presented in the main text. It also contains two additional figures presenting the relative efficiency of inhibiting double resistance emergence for the mixing and cycling strategies (Figures S1 and S2), and a table comparing the mean frequency of overall infected patients, taken over various parameter sets, for all three strategies (Table S1). Click here for file
