# Supplementary material for: Implications of stress-induced genetic variation for minimizing multidrug resistance in bacteria
Source: BMC Med. 2012 Aug 13;10:89. doi: 10.1186/1741-7015-10-89 (PMC3482572; doi:10.1186/1741-7015-10-89)
Supplement: Additional file 1 — Proofs and additional figures and tables. Additional file 1 contains proofs for all the equations presented in the main text. It also contains two additional figures presenting the relative efficiency of inhibiting double resistance emergence for the mixing and cycling strategies (Figures S1 and S2), and a table comparing the mean frequency of overall infected patients, taken over various parameter sets, for all three strategies (Table S1). [file 1741-7015-10-89-S1.PDF]

Supporting Material

1. Defining the emergence rate of double resistant bacteria for HGT:

Define R_i to be the frequency of patients infected with bacteria resistant to antibiotic $i \in \{1,2\}$. When modeling constant probabilities of HGT, the emergence of double resistance, for a treatment strategy U, is proportional to:

$$\mathrm{E_{HGT}}(\mathrm{U}) := \int_{\mathrm{t_0}}^{\mathrm{t_1}} \mathrm{R_1}(\mathrm{U})\mathrm{R_2}(\mathrm{U})\mathrm{dt}$$

for a time interval of length $\mathrm{t_1} - \mathrm{t_0}$. The proportion constant includes the rate of a successful bacterial transmission, and probability of HGT of genetic material coding for antibiotic resistance between the different bacteria.

Let r_s, r_r be the probabilities of receiving genetic material encoding for antibiotic resistance in the presence of antibiotics to which the bacteria are susceptible or resistant, respectively. Similarly, let d_s, d_r be the probabilities of donating genetic material encoding for antibiotic resistance in the presence of antibiotics to which the bacteria are susceptible or resistant, respectively. We will assume that these probabilities are independent of the type of antibiotic the bacteria are resistant to. ϕ will be defined as the time delay of stress-induction; σ will be the relative persistence of double resistant bacteria when having no advantage over single resistant bacteria within the same host; C will be defined as the rate of bacterial transmission between patients (all as defined in the main text).

The term that describes the emergence of double-resistant bacteria for a strategy U will be denoted $\xi_{HGT}(U)$. We will derive the explicit expressions of ξ_{HGT} for each of the three strategies.

The emergence of double resistance in combining is straightforward, since all single resistant bacteria are under antibiotic stress.

(1)

$$\xi_{HGT}(Combining) = Cr_s d_s E_{HGT}(Combining)$$

For the cycling strategy we will define α as the antibiotic cycle length. We can divide the time to $\frac{1}{2}\alpha$ length segments, in which only one drug is used in the hospital unit. After α days the process is repeated and the first antibiotic is applied again. We take $\mathrm{t_0} = 0, \mathrm{t_1} = k\alpha$, for some $\mathrm{k} \in \mathrm{N}$, to avoid bias. Since at each of these segments the antibiotic treatment is constant we get:

$$\xi_{HGT}\left(Cycling\right)=\frac{C}{4}\sum_{i=0}^{2k-1}\int_{\frac{i}{2}\alpha}^{\frac{(i+1)}{2}\alpha}\left(r_r d_s\sigma+r_r d_s+r_s d_r+r_s d_r\sigma\right)R_1\left(\text{cycling}\right)\ R_2\left(\text{cycling}\right)dt$$

Since only one antibiotic is used, one strain of bacteria will be stressed throughout each time segment. The multiplier inside the integral arises from the transportation of bacteria from one patient to another. The double resistant bacteria's probability of contributing to double resistance emergence is changed according to the relative advantage they have over the host's bacterial population (thus the multiplication by σ). Equation (2) is simplified to:

(2)

$$\xi_{HGT}\left(Cycling\right)=\frac{C}{4}\left(r_r d_s\sigma+r_r d_s+r_s d_r+r_s d_r\sigma\right)\sum_{i=0}^{2k-1}\int_{\frac{i}{2}\alpha}^{\frac{(i+1)}{2}\alpha}R_1\left(\text{cycling}\right)\ R_2\left(\text{cycling}\right)dt$$

$$=\frac{C}{4}(r_r d_s+r_s d_r)(1+\sigma)E_{HGT}(cycling)$$

Since mixing is the only strategy in which patients are treated with different antibiotics it has a more complex expression for the double resistance emergence. Under the mixing strategy bacteria can be transported between patients treated with the same or different antibiotics and consequently ϕ now also appears in the dynamics. Therefore, for the mixing strategy we get:

$$\xi_{HGT}\left(mixing\right)=C\int_{t_0}^{t_1}\frac{1}{8}\Big[r_s(d_s\phi+d_r(1-\phi))+r_r d_s\sigma+r_s d_r+r_r(d_r\phi+d_s(1-\phi))\sigma+$$
$$d_s(r_s\phi+(1-\phi)r_r)+d_s r_r+d_r r_s\sigma+d_r(r_r\phi+r_s(1-\phi))\sigma\Big]R_1\left(\text{mixing}\right)R_2\left(\text{mixing}\right)dt$$

Which simplifies to:

(3)

$$\xi_{HGT}\left(Cycling\right)=C\frac{1}{8}\Big[r_s(d_s\phi+d_r(1-\phi))+r_r d_s\sigma+r_s d_r+r_r(d_r\phi+d_s(1-\phi))\sigma+$$
$$d_s(r_s\phi+(1-\phi)r_r)+d_s r_r+d_r r_s\sigma+d_r(r_r\phi+r_s(1-\phi))\sigma\Big]E_{HGT}(Mixing)$$

<u>1.1 Comparing the emergence of double resistance for different strategies under stress-induced HGT</u>

We will compare the influence of stress-induced HGT on the different strategies. Due to the simplifications presented in equations 1-3, we can vary $r_r, r_s, d_r, d_s, \phi, \sigma$ independently of the E_{HGT} value. Thus we can compare ξ_{HGT} for different strategies. Note that we can disregard C throughout this section, since it appears as a constant multiplier in the emergence of double resistant bacteria for all of the strategies.

Mixing Vs. Combining

When comparing mixing to combining we get the following expression:

$$\frac{\xi_{HGT}(mixing)}{\xi_{HGT}(combining)} =$$

$$\frac{\frac{1}{8}\left[r_s(d_s\phi + d_r(1-\phi)) + r_r d_s\sigma + r_s d_r + r_r(d_r\phi + d_s(1-\phi))\sigma + d_s(r_s\phi + (1-\phi)r_r) + d_s r_r + d_r r_s\sigma + d_r(r_r\phi + r_s(1-\phi))\sigma\right]E_{HGT}(mixing)}{r_s d_s E_{HGT}(combining)}$$

Since the dynamics determining the value of E_{HGT} are very complex, we can explore the multiplier of $\frac{E_{HGT}(mixing)}{E_{HGT}(combining)}$ and see if it can have a strong influence on $\frac{\xi_{HGT}(mixing)}{\xi_{HGT}(combining)}$. The term describing the ratio of E_{HGT} coefficients is:

(4)

$$\frac{\frac{1}{8}\left[r_s(d_s\phi + d_r(1-\phi)) + r_r d_s\sigma + r_s d_r + r_r(d_r\phi + d_s(1-\phi))\sigma + d_s(r_s\phi + (1-\phi)r_r) + d_s r_r + d_r r_s\sigma + d_r(r_r\phi + r_s(1-\phi))\sigma\right]}{r_s d_s} =$$

$$\frac{1}{8}\left[\phi + \frac{d_r}{d_s}(1-\phi) + \frac{r_r}{r_s}\sigma + \frac{d_r}{d_s} + \frac{r_r}{r_s}(\frac{d_r}{d_s}\phi + (1-\phi))\sigma + \phi + (1-\phi)\frac{r_r}{r_s} + \frac{r_r}{r_s} + \frac{d_r}{d_s}\sigma + \frac{d_r}{d_s}(\frac{r_r}{r_s}\phi + (1-\phi))\sigma\right] =$$

$$\frac{1}{8}\left[2\phi + \frac{d_r}{d_s}(2-\phi) + \frac{r_r}{r_s}(2-\phi)\sigma + (2-\phi)\frac{r_r}{r_s} + \frac{d_r}{d_s}\sigma(2-\phi) + 2\frac{d_r}{d_s}\frac{r_r}{r_s}\phi\sigma\right] =$$

$$\frac{1}{8}\left[2\phi + \frac{d_r}{d_s}\left[(2-\phi) + \sigma(2-\phi)\right] + \frac{r_r}{r_s}\left[(2-\phi) + (2-\phi)\sigma\right] + 2\frac{d_r}{d_s}\frac{r_r}{r_s}\phi\sigma\right] =$$

$$\frac{1}{8}\left[2\phi + \left(\frac{d_r}{d_s} + \frac{r_r}{r_s}\right)(2-\phi)(1+\sigma) + 2\frac{d_r}{d_s}\frac{r_r}{r_s}\phi\sigma\right]$$

Since $0 \le \phi \le 1, 0 \le \frac{d_r}{d_s} \le 1, 0 \le \frac{r_r}{r_s} \le 1, 0 \le \sigma \le 1$, the upper bound of this term is 1. The lower bound is more complex. It is mainly dominated by $\max\left\{\phi, \frac{d_r}{d_s}, \frac{r_r}{r_s}\right\}$. If the response of the bacteria to stress is quick, i.e. ϕ is small, then the stress-induction mechanisms have a strong effect. In this case $\frac{\xi_{HGT}(mixing)}{\xi_{HGT}(combining)} \le \left[\max\left\{\frac{d_r}{d_s}, \frac{r_r}{r_s}\right\} + \frac{1}{4}((\frac{d_r}{d_s}\frac{r_r}{r_s} + 1)\phi)\right]\frac{E_{HGT}(mixing)}{E_{HGT}(combining)}$.

Therefore for a large enough effect of stress on both donors and recipients of genetic material and a small value of ϕ, $\frac{\xi_{HGT}(mixing)}{\xi_{HGT}(combining)}$ will tend to zero. If the response of the bacteria to stress is slow,

i.e. ϕ larger then $\frac{d_r}{d_s}, \frac{r_r}{r_s}$, then even when stress affects HGT drastically, the coefficient will have a lower bound of $\frac{\phi}{4}$.

Cycling Vs. Mixing

From equations 2 and 3 we know that

$$\frac{\xi_{HGT}(mixing)}{\xi_{HGT}(cycling)} = \frac{\frac{1}{8}\left[r_s(d_s\phi + d_r(1-\phi)) + r_r d_s\sigma + r_s d_r + r_r(d_r\phi + d_s(1-\phi))\sigma + d_s(r_s\phi + (1-\phi)r_r) + d_s r_r + d_r r_s\sigma + d_r(r_r\phi + r_s(1-\phi))\sigma\right]}{\frac{1}{4}(r_r d_s\sigma + r_s d_r\sigma + r_r d_s + r_s d_r)} \frac{E_{HGT}(mixing)}{E_{HGT}(cycling)}$$

We will compare the coefficient of $\frac{E_{HGT}(mixing)}{E_{HGT}(cycling)}$:

(5)

$$\frac{\frac{1}{8}\left[r_s(d_s\phi + d_r(1-\phi)) + r_r d_s\sigma + r_s d_r + r_r(d_r\phi + d_s(1-\phi))\sigma + d_s(r_s\phi + (1-\phi)r_r) + d_s r_r + d_r r_s\sigma + d_r(r_r\phi + r_s(1-\phi))\sigma\right]}{\frac{1}{4}(r_r d_s\sigma + r_s d_r\sigma + r_r d_s + r_s d_r)} =$$

$$= \frac{1}{2}\left[\frac{\left[2\phi + \left(\frac{d_r}{d_s} + \frac{r_r}{r_s}\right)(2-\phi)(1+\sigma) + 2\frac{d_r}{d_s}\frac{r_r}{r_s}\phi\sigma\right]}{\left(\frac{r_r}{r_s} + \frac{d_r}{d_s}\right)(1+\sigma)}\right] =$$

$$\frac{1}{2}\left[\frac{2\phi}{\left(\frac{r_r}{r_s} + \frac{d_r}{d_s}\right)(1+\sigma)} + \frac{2\frac{d_r}{d_s}\frac{r_r}{r_s}\phi\sigma}{\left(\frac{r_r}{r_s} + \frac{d_r}{d_s}\right)(1+\sigma)} + (2-\phi)\right]$$

This term has a lower bound of 1. When $\frac{d_r}{d_s}$ and $\frac{r_r}{r_s}$ decrease, the expression increases. The upper bound is mainly determined by the term : $\frac{2\phi}{\left(\frac{r_r}{r_s} + \frac{d_r}{d_s}\right)(1+\sigma)}$.

Thus $\frac{\xi_{HGT}(mixing)}{\xi_{HGT}(cycling)} \geq \frac{\phi}{2\max\left\{\frac{r_r}{r_s},\frac{d_r}{d_s}\right\}(1+\sigma)}\frac{E_{HGT}(mixing)}{E_{HGT}(cycling)}$ which means that if

$\max\left\{\frac{r_r}{r_s},\frac{d_r}{d_s}\right\} \ll \phi$, and $\max\left\{\frac{r_r}{r_s},\frac{d_r}{d_s}\right\}$ is small enough, $\xi_{HGT}(mixing) \geq \xi_{HGT}(cycling)$. If ϕ is zero then $\frac{\xi_{HGT}(mixing)}{\xi_{HGT}(cycling)} = \frac{E_{HGT}(mixing)}{E_{HGT}(cycling)}$.

Combining Vs. Cycling

From equations 1 and 3 we know that

$$\frac{\xi_{HGT}(combining)}{\xi_{HGT}(cycling)} = \frac{r_s d_s}{\frac{1}{4}(r_r d_s\sigma + r_s d_r\sigma + r_r d_s + r_s d_r)}\frac{E_{HGT}(combining)}{E_{HGT}(cycling)}$$

Again, we will compare the coefficient of $\frac{E_{HGT}(combining)}{E_{HGT}(cycling)}$:

(6)

$$\frac{r_s d_s}{\frac{1}{4}(r_r d_s\sigma + r_s d_r\sigma + r_r d_s + r_s d_r)} = \frac{4}{\left(\frac{r_r}{r_s}+\frac{d_r}{d_s}\right)(1+\sigma)}$$

This term has a lower bound of 1. We can also see that

$$\frac{\xi_{HGT}(combining)}{\xi_{HGT}(cycling)} \geq \frac{1}{\max\left\{\frac{r_r}{r_s},\frac{d_r}{d_s}\right\}}\frac{E_{HGT}(combining)}{E_{HGT}(cycling)}$$

Thus if HGT is influenced enough by stress $\xi_{HGT}(combining) \geq \xi_{HGT}(cycling)$

2. SIM

Analogous to the stress induced HGT, we define μ_r and μ_s as the mutation rates when bacteria are not under antibiotic stress, and when they are under antibiotic stress, respectively.

We will also define the following term, for a treatment strategy U :

$$E_M(U) := \int_{t_0}^{t_1}\left(R_1(U)+R_2(U)\right)dt$$

The emergence of double-resistant bacteria for a strategy U will be denoted $\xi_{SIM}(U)$. We will derive the explicit expressions of ξ_{SIM} for each of the three strategies.

The emergence of double resistance under the mixing strategy will be:

(7)

$$\xi_{SIM}\left(Mixing\right)=\frac{1}{2}\left(\mu_r\sigma+\mu_s\right)E_M(Mixing)$$

For combining, the term describing double resistance emergence will be:

(8)

$$\xi_{SIM}\left(Combining\right)=\mu_s E_M(Combinig)$$

The emergence of double resistance under cycling is a bit messier. If T is the antibiotic currently used in the hospital we get:

$$\xi_{SIM}\left(Cycling\right)=\int_{t_0}^{t_1}\left(R_1\left(\mu_s I_{\{2\in T\}}+\mu_r(1-I_{\{2\in T\}})\sigma\right)+R_2\left(\mu_s I_{\{1\in T\}}+\mu_r(1-I_{\{1\in T\}})\sigma\right)\right)dt$$

We will divide the integral to time segments, as was defined in section 1.

$$\xi_{SIM}\left(Cycling\right)=\sum_{i=0}^{2k-1}\int_{\frac{i}{2}\alpha}^{\frac{(i+1)}{2}\alpha}\left(R_1\left(\mu_s I_{\{2\in T\}}+\mu_r(1-I_{\{2\in T\}})\sigma\right)+R_2\left(\mu_s I_{\{1\in T\}}+\mu_r(1-I_{\{1\in T\}})\sigma\right)\right)dt$$

$$=\sum_{i=0}^{k-1}\int_{i\alpha}^{i\alpha+\frac{1}{2}\alpha}\left(R_1\left(\mu_s I_{\{2\in T\}}+\mu_r(1-I_{\{2\in T\}})\sigma\right)+R_2\left(\mu_s I_{\{1\in T\}}+\mu_r(1-I_{\{1\in T\}})\sigma\right)\right)dt+$$
$$\sum_{i=0}^{k-1}\int_{i\alpha+\frac{1}{2}\alpha}^{i\alpha+\alpha}\left(R_1\left(\mu_s I_{\{2\in T\}}+\mu_r(1-I_{\{2\in T\}})\sigma\right)+R_2\left(\mu_s I_{\{1\in T\}}+\mu_r(1-I_{\{1\in T\}})\sigma\right)\right)dt$$

Simplifying and assuming, without loss of generality, that each cycle starts with treatment with antibiotic 1 we get:

$$\xi_{SIM}\left(Cycling\right)=\sum_{i=0}^{k-1}\int_{i\alpha}^{i\alpha+\frac{1}{2}\alpha}\left(\mu_r R_1\left(Cycling\right)\sigma+\mu_s R_2\left(Cycling\right)\right)dt+\sum_{i=0}^{k-1}\int_{i\alpha+\frac{1}{2}\alpha}^{i\alpha+\alpha}\left(\mu_s R_1\left(Cycling\right)+\mu_r R_2\left(Cycling\right)\sigma\right)dt$$

We will define:

(9)

$$A := \sum_{i=0}^{k-1} \int_{\mathrm{i}\alpha+\frac{1}{2}\alpha}^{\mathrm{i}\alpha+\alpha} R_1\left(Cycling\right)\, dt + \sum_{i=0}^{k-1} \int_{\mathrm{i}\alpha}^{\mathrm{i}\alpha+\frac{1}{2}\alpha} R_2\left(Cycling\right)\, dt$$

$$B := \sum_{i=0}^{k-1} \int_{\mathrm{i}\alpha+\frac{1}{2}\alpha}^{\mathrm{i}\alpha+\alpha} R_2\left(Cycling\right)\, dt + \sum_{i=0}^{k-1} \int_{\mathrm{i}\alpha}^{\mathrm{i}\alpha+\frac{1}{2}\alpha} R_1\left(Cycling\right)\, dt$$

Thus $A+B = \int_{t_0}^{t_1} \left(R_1\left(Cycling\right) + R_2\left(Cycling\right)\right) dt = E_M(Cycling)$

And thus we get:

(10)

$$\sum_{i=0}^{k-1} \int_{\mathrm{i}\alpha}^{\mathrm{i}\alpha+\frac{1}{2}\alpha} \left(\mu_r R_1\left(Cycling\right)\sigma + \mu_s R_2\left(Cycling\right)\right)\, dt + \sum_{i=0}^{k-1} \int_{\mathrm{i}\alpha+\frac{1}{2}\alpha}^{\mathrm{i}\alpha+\alpha} \left(\mu_s R_1\left(Cycling\right) + \mu_r R_2\left(Cycling\right)\sigma\right)\, dt =$$

$$\mu_s \left[\sum_{i=0}^{k-1} \int_{\mathrm{i}\alpha+\frac{1}{2}\alpha}^{\mathrm{i}\alpha+\alpha} R_1\left(Cycling\right)\, dt + \sum_{i=0}^{k-1} \int_{\mathrm{i}\alpha}^{\mathrm{i}\alpha+\frac{1}{2}\alpha} R_2\left(Cycling\right)\, dt \right] +$$

$$\mu_r \sigma \left[\sum_{i=0}^{k-1} \int_{\mathrm{i}\alpha+\frac{1}{2}\alpha}^{\mathrm{i}\alpha+\alpha} R_2\left(Cycling\right)\, dt + \sum_{i=0}^{k-1} \int_{\mathrm{i}\alpha}^{\mathrm{i}\alpha+\frac{1}{2}\alpha} R_1\left(Cycling\right)\, dt \right] =$$

$= \mu_s A + \mu_r \sigma B$.

2.1 SIM: Comparing the emergence of double resistance for the different strategies

If $\boldsymbol{A} \le \boldsymbol{B}$ (A and B as defined in (9)) then:

$\mu_s A + \mu_r \sigma B \le \frac{1}{2}\left(\mu_s + \mu_r \sigma\right)\left(A+B\right) \Leftrightarrow$

$\frac{1}{2}\mu_s A+\frac{1}{2}\mu_r\sigma B\le\frac{1}{2}\mu_s B+\frac{1}{2}\mu_r\sigma A\Leftrightarrow$

$\mu_s\left(A-B\right)\le\mu_r\sigma\left(A-B\right)\Leftrightarrow$

$\mu_s\ge\mu_r\sigma$ which is true under mutation is stress-induced.

$\boldsymbol{A}\le\boldsymbol{B}$ is usually true since the frequency of a resistant strain is lower when the drug applied is the one it is susceptible to. We have also verified this by simulating random parameter sets and observing the values of A and B (results not shown).

Thus, assuming $\boldsymbol{A}\le\boldsymbol{B}$ and using inequality (18) and $\mu_s\ge\mu_r$, we get:

$$\xi_{SIM}\left(Cycling\right)=\mu_s A+\mu_r\sigma B\le\frac{1}{2}(\mu_s+\mu_r\sigma)\left(A+B\right)\le\mu_s(A+B)$$

Now we can see that:

(11)

$$\frac{\xi_{SIM}(Cycling)}{\xi_{SIM}(Mixing)}=\frac{\int_{t_0}^{t_1}\left(R_1\left(Cycling\right)\left(\mu_s \mathrm{I}_{\{2\in T\}}+\mu_r(1-\mathrm{I}_{\{2\in T\}})\sigma\right)+R_2\left(Cycling\right)\left(\mu_s \mathrm{I}_{\{1\in T\}}+\mu_r(1-\mathrm{I}_{\{1\in T\}})\sigma\right)\right)\,dt}{\frac{1}{2}\left(\mu_r\sigma+\mu_s\right)\int_{t_0}^{t_1}\left(R_1\left(Mixing\right)+R_2\left(Mixing\right)\right)\,dt}\le$$

$$\le\frac{\frac{1}{2}\left(\mu_s+\mu_r\sigma\right)E_M(Cycling)}{\frac{1}{2}\left(\mu_r\sigma+\mu_s\right)E_M(Mixing)}=\frac{E_M(Cycling)}{E_M(Mixing)}$$

And

(12)

$$\frac{\xi_{SIM}(Cycling)}{\xi_{SIM}(Combining)}=\frac{\int_{t_0}^{t_1}\left(R_1\left(Cycling\right)\left(\mu_s \mathrm{I}_{\{2\in T\}}+\mu_r(1-\mathrm{I}_{\{2\in T\}})\sigma\right)+R_2\left(Cycling\right)\left(\mu_s \mathrm{I}_{\{1\in T\}}+\mu_r(1-\mathrm{I}_{\{1\in T\}})\sigma\right)\right)\,dt}{\mu_s\int_{t_0}^{t_1}\left(R_1\left(Combining\right)+R_2\left(Combining\right)\right)\,dt}\le$$

$$\le\frac{\mu_s E_M(Cycling)}{\mu_s E_M(Combining)}=\frac{E_M(Cycling)}{E_M(Combining)}$$

And of course

(13)

$$\frac{\xi_{SIM}(Mixing)}{\xi_{SIM}(Combining)}=\frac{\frac{1}{2}(\mu_s+\mu_r\sigma)E_M(Mixing)}{\mu_s E_M(Combining)}\le\frac{E_M(Mixing)}{E_M(Combining)}$$

Thus we get the same direction of the effect of stress induced genetic variation on the effectiveness of the strategies as we got in both forms of stress induced HGT.

As for lower bounds on the ratio, we can denote $\mu_r = x$, and $\mu_s = ax$ for $a \geq 1$. Now we see that for A and B as in (9):

(14)

$$\frac{\xi_{SIM}(Cycling)}{\xi_{SIM}(Mixing)} = \frac{\mu_s A + \mu_r \sigma B}{\frac{1}{2}\left(\mu_r \sigma + \mu_s\right)\int_{t_0}^{t_1}\left(R_1\left(Mixing\right) + R_2\left(Mixing\right)\right)dt} =$$

$$\frac{axA + x\sigma B}{\frac{1}{2}\left(x\sigma + ax\right)\int_{t_0}^{t_1}\left(R_1\left(Mixing\right) + R_2\left(Mixing\right)\right)dt} =$$

$$\frac{aA + \sigma B}{\frac{1}{2}\left(\sigma + a\right)E_m(Mixing)} = \left(\frac{aA + \sigma B}{\frac{1}{2}\left(\sigma + u\right)a(A+B)}\right)\frac{A+B}{E_M(Mixing)} = \left(\frac{aA + \sigma B}{\frac{1}{2}\left(\sigma + a\right)(A+B)}\right)\frac{E_M(Cycling)}{E_M(Mixing)}$$

When we take the limit of this expression for $a \to \infty$ we get that the multiplier goes to $\frac{2A}{A+B}$. This means that the effect of SIM on the effectiveness of mixing relative to cycling is bounded by the ratio $\frac{2A}{A+B}$. This ratio expresses the mean frequency of patients infected with single resistant bacteria when they are treated with a drug they are resistant to, relative to the mean of their frequency through time. Note that for the special case of $\sigma = 0$ stress induction does not affect cycling. We will have

$$\left(\frac{A}{\frac{A+B}{2}}\right)\frac{E_M(Cycling)}{E_M(Mixing)}$$

for any amount of stress induction.

With combining the result is similar:

(15)

$$\frac{\xi_{SIM}(Cycling)}{\xi_{SIM}(Combining)} = \left(\frac{aA + \sigma B}{a(A+B)}\right)\frac{E_M(Cycling)}{E_M(Combining)}$$

When we take the limit of this expression for $a \to \infty$, or $\sigma = 0$ for any value of a , the multiplier goes to $\frac{A}{A+B}$. Using (14) and (15) we can easily see that the lower bound on the effect of SIM on the effectiveness of mixing relative to combining is half.

3. Robustness

In order to talk about the robustness of our results to changes in parameters, we will look at the behavior of the frequency of patients with resistant bacteria at equilibrium. We can define a new variable in our system of differential equations: $R := R_1 + R_2$, where $\lambda_R := \lambda_{R_1} + \lambda_{R_2}$. This reduces the order of the polynomial we need to solve for to obtain the equilibrium values.

For the combining strategy the equilibrium reduces to a quadratic equation, where the solutions are

$$S \to \frac{\lambda s(\beta\lambda_R - \gamma\lambda_R - \lambda_R m - \lambda_R \tau \pm \sqrt{\lambda_R{}^2(4\beta(\lambda_R + \lambda s)m + (-\beta + \gamma + m + \tau)^2)})}{2\beta\lambda_R(\lambda_R + \lambda s)}$$

$$R \to \frac{\beta\lambda_R - \gamma\lambda_R - \lambda_R m - \lambda_R \tau \pm \sqrt{\lambda_R{}^2(4\beta(\lambda_R + \lambda s)m + (-\beta + \gamma + m + \tau)^2)}}{2\beta(\lambda_R + \lambda s)}$$

The only solution which is positive (S and R are frequencies) is

$$S \to \frac{\lambda s\left(\beta - \gamma - m - \tau + \sqrt{(4\beta(\lambda r + \lambda s)m + (\beta - \gamma - m - \tau)^2)}\right)}{2\beta(\lambda r + \lambda s)}$$

$$R \to \frac{\lambda_R\left(\beta - \gamma - m - \tau + \sqrt{(4\beta(\lambda_R + \lambda s)m + (\beta - \gamma - m - \tau)^2)}\right)}{2\beta(\lambda_R + \lambda s)}$$

Moreover, we can see that $R_{eq} = S_{eq}\frac{\lambda_R}{\lambda_S}$.

Now we want to find the values of R_1 and R_2 at equilibrium.

At equilibrium we have $\frac{dR_i}{dt} = 0$, therefore:

$$R_i = \frac{\lambda_{R_i} m}{(\tau + \gamma + m - \beta X)}$$

We also know that $X = 1 - R - S = 1 - S\frac{\lambda_R}{\lambda_S} - S = 1 - S(1 + \frac{\lambda_R}{\lambda_S})$

And thus $R_i = \frac{\lambda_{R_{i1}} m}{(\tau + \gamma + m - \beta(1 - S(1 + \frac{\lambda_R}{\lambda_S})))} = \frac{\lambda_{R_i} m}{(\tau + \gamma + m - \beta(1 - S(1 + \frac{\lambda_{R_1} + \lambda_{R2}}{\lambda_S})))}$

And since S is independent of the ratio, we can see that λ_{R_i} (with a constant sum $\lambda_{R_1}+\lambda_{R_2}$) affects the equilibrium of R_i linearly with a slope of one. In addition, $\frac{R_1}{R_2}=\frac{\lambda_{R_1}}{\lambda_{R_2}}$.

We can analyze the equilibrium in the same manner with mixing, however the expression for the equilibrium values there is very long. Suffice to say that we can find a solution for the frequency of X that does not depend on the ratio $\frac{\lambda_{R_1}}{\lambda_{R_2}}$ but only the sum $\lambda_{R_1}+\lambda_{R_2}$ (since we only used λ_R) and once more get that the equilibrium values $R_i=\frac{\lambda_{R_i}m}{(\tau+\gamma+m-\beta X)}$ are dependent on λ_{R_i} in the same way as in combining.

For cycling, in times $i\alpha+\frac{1}{2}\alpha\le t\le i\alpha+\alpha$ antibiotic 2 is used $(T=2)$ and in times $i\alpha+\alpha\le t\le i\alpha+\frac{1}{2}\alpha$ antibiotic 1 is used $(T=1)$ for $i\in N$. The frequencies of the bacterial strains after the drug has been switched and the system has stabilized are:

$$R_1(t)=\begin{cases}\dfrac{\lambda_{R_1}m}{(\tau+\gamma+m-\beta X)}, & i\alpha+\frac{1}{2}\alpha\le t\le i\alpha+\alpha\\ \dfrac{\lambda_{R_1}m}{(\gamma+m-\beta X)}, & i\alpha+\alpha\le t\le i\alpha+\frac{1}{2}\alpha\end{cases}$$

$$R_2(t)=\begin{cases}\dfrac{\lambda_{R_2}m}{(\tau+\gamma+m-\beta X)}, & i\alpha+\alpha\le t\le i\alpha+\frac{1}{2}\alpha\\ \dfrac{\lambda_{R_2}m}{(\gamma+m-\beta X)}, & i\alpha+\frac{1}{2}\alpha\le t\le i\alpha+\alpha\end{cases}$$

Analogously to the other strategies, we can rewrite the equations so the value of X at equilibrium will not be dependent on $\frac{\lambda_{R_1}}{\lambda_{R_2}}$. Thus, if equilibrium is reached, we again have linear dependence on resistant strains entrance rates for a constant entrance rate sum. For our cycling lengths (200 days for each cycle) the system reached equilibrium approximately 10 days after a drug switch.

We have explored the interactions between parameters in the SIM model as well. As we have shown above, the bound on the effect of stress on mutation in cycling depends on the number of patients with resistant bacteria treated with effective antibiotics, relative to all the resistant patients, namely $\left(\frac{A}{A+B}\right)$. By simulating 10^5 random sets of parameters we saw that one of the most influential parameters affecting $\frac{A}{A+B}$ is τ. The intuitive explanation is that as clearance rate due to antibiotic usage increases, the fraction of patients currently treated will quickly reduce. However, we must

note that the value of τ does not affects the values of E_{SIM} for different strategies in the same manner.

<u>Figure S1.</u> Double resistance emergence ratio under mixing and cycling when assuming SIM.

The ratio of double resistance emergence under mixing to double resistance emergence under cycling, ($\frac{\xi_{SIM}(mixing)}{\xi_{SIM}(cycling)}$), is shown as a function of mutation increase under stress, $\frac{\mu_s}{\mu_r}$, and of antibiotic clearance time τ (panel A) or entrance ratio, $\frac{\lambda_{m_1}}{\lambda_{m_2}}$ (panel B). We can see that for a wide range of parameters, especially when mutation rates are strongly increased by stress, the ratio is substantially higher than one (any color different than dark blue in the figure), meaning that cycling is a better strategy for inhibiting double resistance. The emergence is taken over 600 days. The length of each cycle in the cycling strategy is 200 days. Other parameter values (except when varied):

$$\beta = 0.9,\ \gamma = 0.03,\ m = 0.1,\ \lambda_s = 0.1,\ \lambda_{R_1} = 0.1,\ \lambda_{R_2} = 0.1,\ \tau = 0.5, \sigma = 0.5.$$

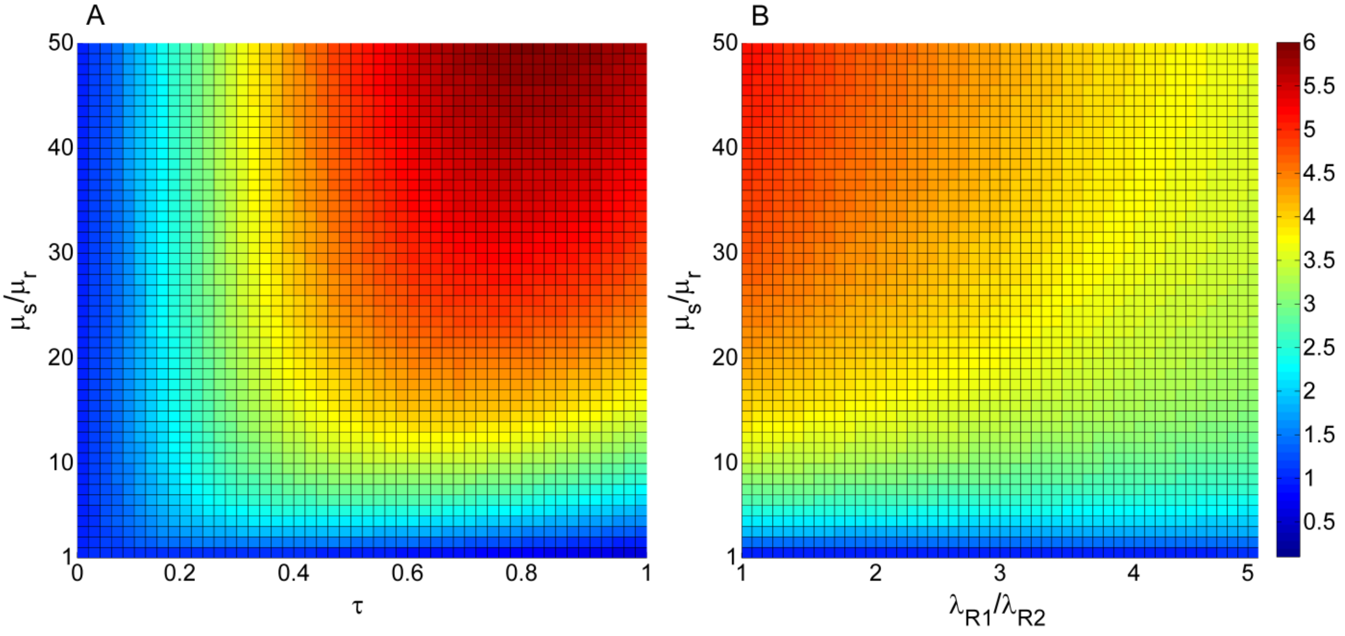

<u>Figure S2.</u> Double resistance emergence ratio under mixing and cycling when assuming stress-induced HGT.

The ratio of double resistance emergence under mixing to double resistance emergence under cycling ($\frac{\xi_{HGT}(mixing)}{\xi_{HGT}(cycling)}$) is plotted as a function of HGT increase under stress, θ, and of antibiotic clearance time τ (panel A) or entrance ratio, $\frac{\lambda_{m_1}}{\lambda_{m_2}}$ (panel B). We can see that for a wide range of parameters, especially when HGT rates are strongly increased by stress, the ratio is substantially higher than one (any color different than dark blue in the figure), meaning that cycling is a better strategy for inhibiting double resistance. The emergence is taken over 600 days. The length of each cycle in the cycling strategy is 200 days. Other parameter values (except when varied):

$$\beta = 0.9,\ \gamma = 0.03,\ m = 0.1,\ \lambda_s = 0.1,\ \lambda_{R_1} = 0.1,\ \lambda_{R_2} = 0.1,\ \tau = 0.5, \sigma = 0.5, \phi = 0.5\,.$$

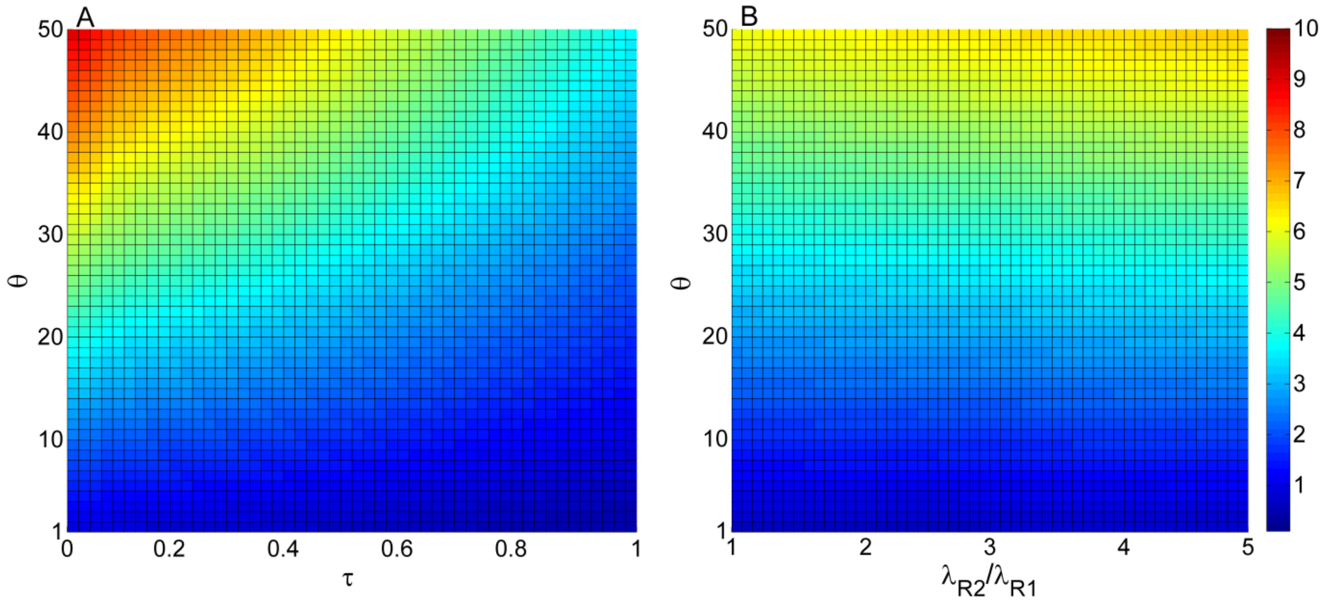

Table S1

The mean values of the proportion of infected patients, emergence of double resistance through mutation, and emergence of double resistance through HGT are given. The means are taken over 10^4 random sets of parameters (the sampling distributions are given in the main text).

	Infection	SIM: $\sigma=0.1$ $\frac{\mu_s}{\mu_r}=1$	SIM: $\sigma=1$ $\frac{\mu_s}{\mu_r}=1$	SIM: $\sigma=0.1$ $\frac{\mu_s}{\mu_r}=10$	SIM: $\sigma=1$ $\frac{\mu_s}{\mu_r}=10$	HGT: $\sigma=0.1$ $\theta=1$	HGT: $\sigma=1$ $\theta=1$	HGT: $\phi=0$ $\theta=10$	HGT: $\phi=1$ $\theta=10$
Cycling	0.73238	$2.6699\cdot10^{-7}$	$4.9089\cdot10^{-7}$	$2.4460\cdot10^{-6}$	$2.6699\cdot10^{-6}$	$2.9803\cdot10^{-14}$	$5.4187\cdot10^{-14}$	$4.0640\cdot10^{-13}$	$4.0640\cdot10^{-13}$
Mixing	0.73226	$2.6992\cdot10^{-7}$	$4.9077\cdot10^{-7}$	$2.4784\cdot10^{-6}$	$2.6992\cdot10^{-6}$	$2.9796\cdot10^{-14}$	$5.4174\cdot10^{-14}$	$4.0630\cdot10^{-13}$	$1.5643\cdot10^{-12}$
Combining	0.72580	$4.8427\cdot10^{-7}$	$4.8427\cdot10^{-7}$	$4.8427\cdot10^{-6}$	$4.8427\cdot10^{-6}$	$5.2766\cdot10^{-14}$	$5.2766\cdot10^{-14}$	$5.2766\cdot10^{-12}$	$5.2766\cdot10^{-12}$
